# Expression Dynamics of Heme Oxygenase-1 in Tumor Cells and the Host Contributes to the Progression of Tumors

**DOI:** 10.3390/jpm11121340

**Published:** 2021-12-09

**Authors:** Jun Fang, Rayhanul Islam, Shanghui Gao, Cheng Zhang, Ryotaro Kunisaki, Shogo Sakaguchi, Naoya Honda, Jian-Rong Zhou, Kazumi Yokomizo

**Affiliations:** 1 Laboratory of Microbiology, Faculty of Pharmaceutical Sciences, Sojo University, Kumamoto 860-0082, Japan; rayhanulislam88@gmail.com (R.I.); gaoshanghui94@gmail.com (S.G.); g1551043@m.sojo-u.ac.jp (R.K.); g1551052@m.sojo-u.ac.jp (S.S.); g1551107@m.sojo-u.ac.jp (N.H.); zhoujr@ph.sojo-u.ac.jp (J.-R.Z.); yoko0514@ph.sojo-u.ac.jp (K.Y.); 2 Department of Toxicology, School of Public Health, Anhui Medical University, Hefei 230032, China; zhangcheng@ahmu.edu.cn

**Keywords:** heme oxygenase-1, carbon monoxide, tumor progression, carcinogenesis

## Abstract

Heme oxygenase (HO-1) plays an important role in cellular protection against various stresses. The induction of HO-1 is an effective strategy for reactive oxygen species-related diseases, inflammatory diseases, as well as suppressing carcinogenesis. On the other hand, the high expression of HO-1 is now well known in many tumors. In this study, we investigated the dynamics of HO-1 expression in the host and the tumor. In the mouse sarcoma S180 solid tumor model and the rat hepatoma AH136B ascitic tumor model, HO-1 expression in the tumor, as indicated by the end product of HO-1 activation, i.e., carbon monoxide, gradually increased along with tumor growth. Over-expression of HO-1 expression in mouse colon cancer C26 tumor cells significantly promoted tumor growth as well as lung metastasis, whereas opposite results were found when the HO-1 expression was reduced in the cells. On the other hand, upregulating HO-1 levels in the host by using an HO-1 inducer protected the progression of the xenograft tumor in mice, whereas lowering HO-1 expression in the host with an HO-1 inhibitor showed accelerated tumor growth and lung metastasis after subcutaneous tumor xenograft inoculation. These findings strongly suggest that the balance of HO-1 levels in the host and the tumor cells is essential for the occurrence, progression, and prognosis of cancer. Maintenance of appropriately high HO-1 levels in the host is favorable for cancer prevention, whereas suppression of HO-1 in the tumor cells may thus become a therapeutic strategy for cancer.

## 1. Introduction

**Figure 1:**
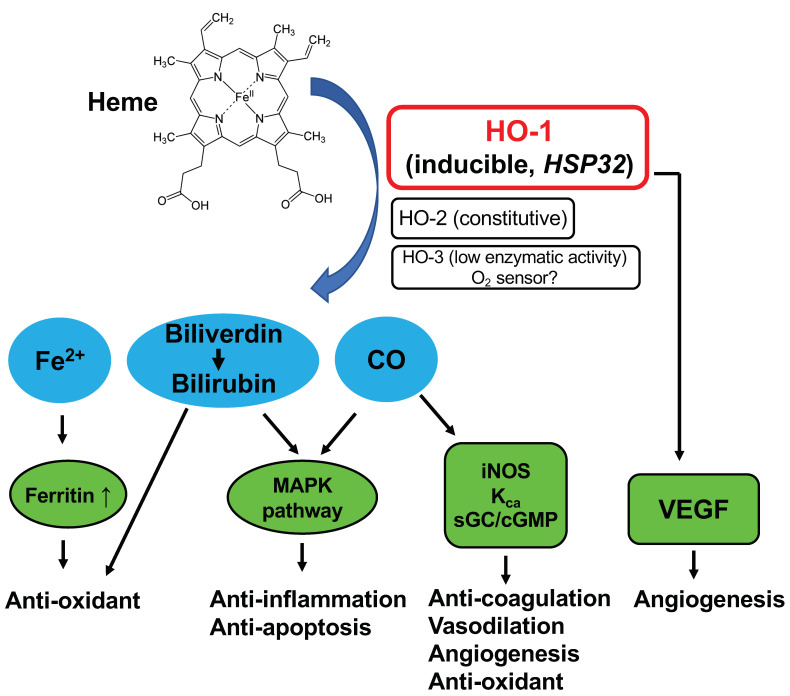
Schematic illustration of heme oxygenase (HO)-catalyzed heme degradation and the roles of HO.

Heme oxygenase (HO) catalyzes the rate-limiting step in the degradation of heme to produce biliverdin, carbon monoxide (CO), and free iron (Fe^2+^) (Figure 1). The biliverdin thus formed is subsequently converted into bilirubin, a potent antioxidant, by cytosolic biliverdin reductase [1,2,3]. The constitutive isoform of HO (HO-2) is highly expressed in the testis and brain under physiological conditions [2]. HO-3 is an isoform detected in many organs, however, its enzyme activity is very low, and the physiological function remains unclear [4]. HO-1, an inducible isoform of HO, is found at low levels in most mammalian tissues but is highly expressed in the liver and spleen [5]. The expression of HO-1 is induced by a wide variety of stress-inducing stimuli, including heat shock [6], ultraviolet irradiation [7], hydrogen peroxide (H_2_O_2_) [7], heavy metals [7,8], hypoxia [9], and nitric oxide (NO) [10,11].

HO-1 may serve a key biological role in the fundamental adaptive and/or defensive response against oxidative stress and cellular stress (Figure 1). Inhibition of HO-1 by using specific HO inhibitors, such as zinc protoporphyrin (ZnPP) or tin protoporphyrin (SnPP), deteriorates the extent of diseases involving the above-mentioned stress stimuli, such as graft rejection [12], ischemia-reperfusion injury [13], cisplatin nephrotoxicity [14], and endotoxin-induced septic shock [15]. In contrast, HO-1 inducers, such as cobalt protoporphyrin (CoPP), or selective over-expression of HO-1 showed beneficial effects on cellular protection in cultured cells and a variety of animal models, including brain, heart, kidney, lung, and liver failure [13,14,16]. Thus, high HO-1 expression may contribute to lowering the occurrence of cancer by protecting healthy cells against various deleterious stresses, including reactive oxygen species (ROS). On the contrary, it has been shown that several human tumors, including brain tumors [17], prostate tumors [18], renal cell carcinoma [19], and oral squamous cell carcinoma [20], express a high level of HO-1. We also found high HO-1 expression in experimental solid tumors, i.e., rat hepatoma AH136B [11] and mouse sarcoma S180 [21,22]. This level of HO-1 expression was comparable to that in the spleen and liver [11,21,22]. Administration of the HO inhibitor ZnPP via a tumor-feeding artery significantly suppressed the growth of AH136B tumors, which suggests a vital role of HO-1 in tumor growth [11]. Though many studies showed the beneficial effect of HO-1 in tumor growth, recent studies also suggest an opposite function of HO-1 in cancer cells, where cancer cells could be killed by the application of HO-1 or its product CO, probably via multiple mechanisms, including the induction of ferroptosis, induction of metabolic exhaustion, and apoptosis of the cancer cells [23,24]. Conversely, the induction of ferroptosis by excessive HO-1 may also trigger detrimental effects in many diseases [23]. These findings suggested the dual roles of HO-1 as a double-edged sword in different cells and different disease conditions. In this context, it is important to investigate the dynamics and balance of HO-1 expression in the healthy cells of the host and in tumor cells, which can be a determinant of carcinogenesis.

Among the enzymatic products of heme by HO-1, CO has been suggested to be an important molecule involved in the cytoprotective mechanism of HO-1 [16]. Exogenous administration of CO inhibits apoptosis in various cells, including fibroblasts, endothelial cells, and vascular smooth muscle cells [25,26,27]. Moreover, a low concentration of CO can protect against hyperoxic lung injury in vivo [28]. One of our recent studies showed that circulating CO concentration is parallel with the HO-1 activity and expression in solid tumors, both in mouse solid tumor models and in human cancer patients, which is positively associated with the growth, metastasis, and malignancy of cancer [29]. A potent antioxidant, canolol, remarkably suppressed the carcinogenesis in an azoxymethane (AOM)/Dextran sulfate sodium (DSS)-induced mouse colon cancer model, in which the HO-1 expression in colon tissues containing tumor nodules was significantly downregulated [30]. Conversely, another study using canolol showed significantly increased HO-1 expression in human retinal pigment epithelial cells [31]. These findings indicated the different expression profiles of HO-1 in normal tissues and tumor tissues during the initiation and progression of cancer. Namely, HO-1 protects healthy tissues against carcinogen-induced injury, but once the tumors form, it will favor their growth and progression.

To elucidate the balance of the host/tumor HO-1 expression and its impact on tumor progression, in this study, we used mouse colon cancer cells with the stable over-expression or knock-down of HO-1 to compare their potentials to form solid tumors in mice. On the other hand, we used an HO-1 inducer or inhibitor to trigger or suppress the expression of HO-1 in the host mice and compared the growth of tumors in these mice. Furthermore, we confirm and highlight the association of CO concentrations and tumor progression in different tumor models.

## 2. Materials and Methods

### 2.1. Materials

ZnPP hemin was purchased from Sigma Chemical Co. (St. Louis, MO, USA). H_2_O_2_ was purchased from Wako Pure Chemical (Osaka, Japan). Pirarubicin hydrochloride was from NIPPON KAYAKU Inc. (Tokyo, Japan). The poly (ethylene glycol) (NOF, Tokyo, Japan)-conjugated ZnPP and hemin, i.e., PEG-ZnPP and PEG-hemin, respectively, were prepared as described previously [21,32]. NOC-7 was kindly provided by Dojin Chemical Ltd., Tokyo, Japan. Other reagents were of reagent grade and were used without further purification.

### 2.2. Cells

Mouse colon cancer C26 cells were kindly provided by Dr Yu Ishima of Tokushima University, Japan. C26 cells with HO-1 over-expression and HO-1 knock-down were established by Shanghai GenePharm Co., Ltd., (Shanghai, China). Briefly, HO-1 cDNA or HO-1 shRNA was transfected into C26 cells using lentivirus, and the stably transfected cells were selected to obtain HO-1 over-expressed C26 cell line (V8971), HO-1 knock-down C26 cell line (6708), respectively; C26 cells transfected by empty lentivirus were used as the vehicle (3NC).

### 2.3. Animals

Male ddY mice and Balb/c mice, all 6 weeks old and each weighing 30–35 g, and male Donryu rats weighing 160–180 g were from SLC, Inc. (Shizuoka, Japan). All animals were maintained at 22 ± 1 °C and 55 ± 5% relative humidity with a 12 h light/dark cycle. All experiments were approved by the Animal Ethics Committees of Sojo University (no. 2020-P-009, approved on 1 April 2020) and carried out according to the Laboratory Protocol for Animal Handling of Sojo University.

### 2.4. Tumor Model

Rat AH136B (rat hepatoma) ascitic tumor was obtained by implanted AH136B tumor cells intraperitoneally (i.p.) into Donryu rats with an inoculum size of 2 × 10^7^ cells. Solid S180 tumor was obtained by inoculated mouse sarcoma S180 cells (2 × 10^6^) subcutaneously (s.c.) in the dorsal skin of ddY mice. Mouse colon cancer C26 cells were maintained by in vitro culture using RPMI-1640 medium (Wako), supplemented with 10% fetal bovine serum (Nichirei Biosciences Inc., Tokyo, Japan) under 5% CO_2_/air at 37 °C. The cultured cells were collected and suspended in physiological saline at a concentration of 2 × 10^7^ cells/mL, and 0.1 mL of cell suspension was implanted into the dorsal skin of Balb/c mice to obtain the C26 tumor model. 

### 2.5. Quantification of CO in the Blood in Mice Bearing Tumors

Rats or mice with AH136B ascitic tumor or S180 solid tumor were killed after scheduled times, and the blood was collected from the tail vein or inferior vena cava. The collected blood was then diluted serially by PBS (-) (samples subjected to this test were at least diluted two times by PBS (-)) and was put in the 10 mL glass test tube on ice with a volume of 4 mL. Then, the test tubes were sealed, and the air in the test tubes was substituted by purged nitrogen gas, after which NOC-7 was added to the test tubes with a final concentration of 1 mM. After a scheduled period of incubation (e.g., 2 h) at room temperature, 1 mL of the gas in the test tubes was collected, which was subjected to (TRIlyzer mBA-3000; Taiyo Instruments, Inc., Osaka, Japan) for CO quantification. 

### 2.6. Cell Viability Assay

Wide C26 cells (transfected with lentivirus, vehicle), as well as C26 cells with HO-1 over-expression and HO-1 knock-down, were seeded in 96-well plates (3000 cells/well). After overnight incubation, H_2_O_2_ or pirarubicin of different concentrations was added, and the cells were cultured for 48 h. Then, cell viability was measured by MTT assay [33]. 

### 2.7. Effect of HO-1 Inducer (PEG-hemin) and HO-1 Inhibitor (PEG-ZnPP) on the Growth of C26 Solid Tumor

A mouse colon cancer C26 solid tumor model using wild type C26 cells was used in this study. One week before inoculation of C26 cells into mice, the mice were treated with PEG-hemin or PEG-ZnPP, both at 5 mg/kg (hemin or ZnPP equivalent) by intravenous injection (i.v.) thrice a week, which continued to 1 week after tumor inoculation. The tumor volume (mm^3^), which was calculated as (W^2^ × L)/2 by measuring the width (W) and length (L) of the tumor, was recorded every 2–3 days during the period of the experiment. When the tumor reached the size of 4000 mm^3^, the mice were euthanized. At 40 days after tumor inoculation, all the mice were killed, and the lungs were collected. Tumor nodules in the lung were identified macroscopically, and numbers of metastatic nodules and each diameter were counted.

In a separate experiment, healthy Balb/c mice were treated with PEG-hemin or PEG-ZnPP in the above-described protocol, after which the CO concentrations in the blood of mice were measured by the same method as described above. 

### 2.8. In Vivo Tumor Growth of C26 Cells with Different Expression Levels of HO-1 in Balb/c Mice

Wide C26 cells (transfected with lentivirus, vehicle), as well as C26 cells with HO-1 over-expression and HO-1 knock-down, were used in this study to establish solid tumors in Balb/c mice as described above. Tumor volume, as well as lung metastasis, was measured by the same protocol as shown above. 

### 2.9. Statistical Analysis

All data are expressed as means ± standard error of the mean (SEM). Data were analyzed by using ANOVA followed by the Bonferroni correction. A difference was considered statistically significant when *p* < 0.05.

## 3. Results

### 3.1. CO Production in the Ascite and Blood of AH136B Ascitic Tumor-Bearing Rat, and Its Association with Tumor Growth

Doi et al. have reported that rat hepatoma AH136B solid tumors highly expressed HO-1 [11]. So, we first investigated the CO levels in the blood of AH136B ascitic tumor-bearing rats at different stages of tumor progress. As shown in supplemental data Figure S1A, CO production in the blood was detected after 1 h incubation with NO, and it reached a plateau after 2 h incubation with NO. More importantly, a linear change was observed for this gas chromatographic method when the blood was diluted serially (supplemental data Figure S1B), which suggests the liability of this method for CO measurement.

**Figure 2:**
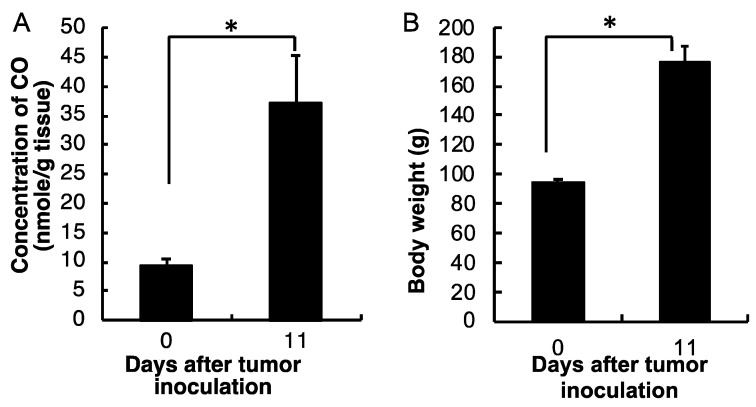
Dynamic changes in CO concentrations in the blood of the AH136B ascitic tumor-bearing rat (**A**), and the tumor growth of AH136B ascitic tumor as described by the body weight changes (**B**). Data are mean ± SEM, *n* = 4–6. * *p* < 0.05. See text for details.

We further measured the CO production in the blood of the AH136B ascitic tumor-bearing rat to see if it increases along with the tumor growth. As shown in Figure 2A, CO production in the blood of the AH136B ascitic tumor-bearing rat remarkably increased on the 11th day after tumor i.p. inoculation, at which point the tumor became considerably aggressive, as reflected by the change in body weight (Figure 2B) compared with that at day 0 (before tumor inoculation) (*p* = 0.028). This result indicates that CO concentration in blood is positively associated with tumor growth. Because CO in the body is mostly the consequence of HO-1-triggered heme metabolism [1,2], and our previous study clearly showed the increased circulating CO is parallel with the upregulation of HO-1 [29], these findings indicated increased HO-1 expression during tumor growth.

### 3.2. Increasing Blood CO Production in S180 Solid Tumor-Bearing Mice Accompanied with Tumor Growth

**Figure 3:**
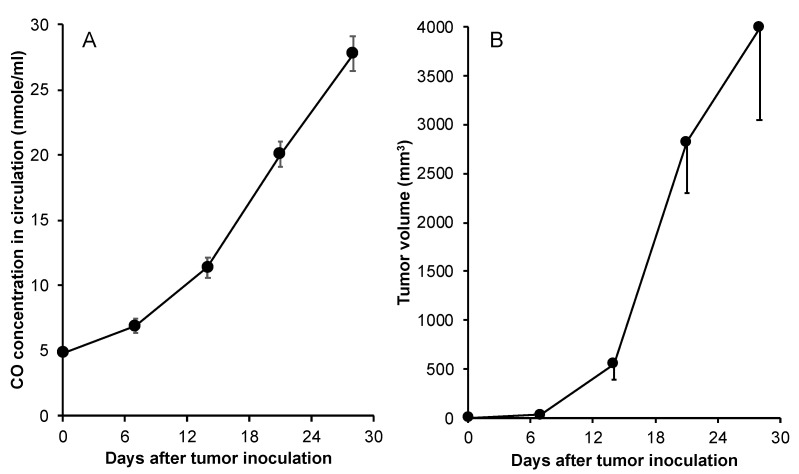
CO production in the mouse sarcoma S180 tumor model. In mouse sarcoma S180 tumor model, after tumor inoculation, mice were killed, and blood was collected. The CO concentrations in the blood were then measured by gas chromatography according to the protocol described in the text (**A**). Meanwhile, the sizes of tumors were measured and tumor volumes were calculated (**B**). Data are mean ± SEM, *n* = 4–6.

To further clarify if the CO production derived from HO-1 in blood accompanied by tumor growth is a universal phenomenon, we further investigated the circulating CO in a mice sarcoma S180 solid tumor model. As shown in Figure 3A, CO production in the blood of S180 tumor-bearing mice started to increase from 7 days after tumor implantation and kept increasing, which was parallel with the tumor growth (Figure 3B).

### 3.3. HO-1 Expression Contributes to the Vulnerability of C26 Cells to ROS and the Anticancer Drug

**Figure 4:**
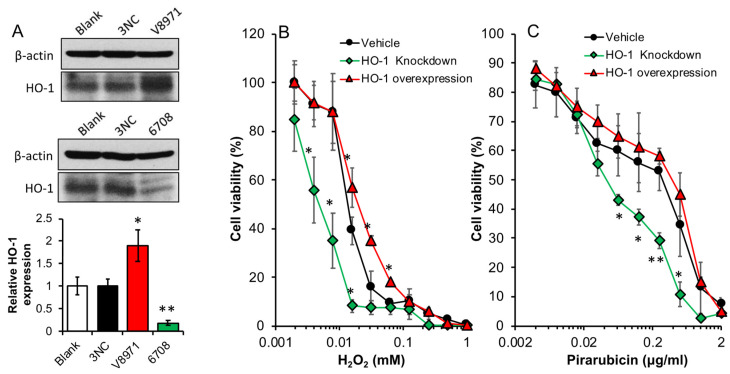
Cytotoxicity of hydrogen peroxide and anticancer drug pirarubicin against colon cancer C26 cells with different expression levels of HO-1. Colon cancer C26 cells transfected with HO-1 (V8971) or HO-1 shRNA (6708) to over-express or knock down HO-1 in the cells, which showed significantly increased or decreased HO-1 expression, compared to C26 cells transfected with empty vector (3NC) (**A**). To each cell line, hydrogen peroxide (**B**) or anticancer drug pirarubicin (**C**) was added and the cells were treated for 48 h, followed by the MTT assay. Data are mean ± SEM, *n* = 3 for (**A**), and *n* = 6–8 for (**B**,**C**). * *p* < 0.05; ** *p* < 0.01 vs. vehicle or blank. See text for details.

To clarify the effect of HO-1 expression on the survival and growth of the tumor cells, we established HO-1 over-expressed C26 cells (V8971) as well as HO-1 knock-down C26 cells (6708), which a 1.8-fold increased HO-1 expression, and an 80% decrease in HO-1 expression were achieved, respectively, compared to the vehicle C26 cells (3NC) (Figure 4A). The cytotoxicities of hydrogen peroxide (H_2_O_2_) and the anticancer drug pirarubicin, which is an anthracycline antibiotic agent similar to doxorubicin that generates ROS as one of the major anticancer mechanisms, to these cells were measured and compared. As shown in Figure 4B, dose-dependent cytotoxicity of H_2_O_2_ was observed, and more importantly, knock-down of HO-1 resulted in a higher vulnerability of cells to H_2_O_2_ (IC_50_ of 0.005 mM vs. 0.018 mM of vehicle cells), whereas HO-1 over-expression gave the cells a slightly increased resistance to this treatment (IC_50_ of 0.025 mM vs. 0.018 mM of vehicle cells). Similar results were also found when pirarubicin was used (Figure 4C). In this study, we used HO-1 knock-down cells. However, HO-1 knock-out cells may exhibit similar characteristics, i.e., increased vulnerability to ROS or ROS-inducing agents [34].

### 3.4. HO-1 Expression Contributes to the Growth and Metastasis of C26 Solid Tumors

**Figure 5:**
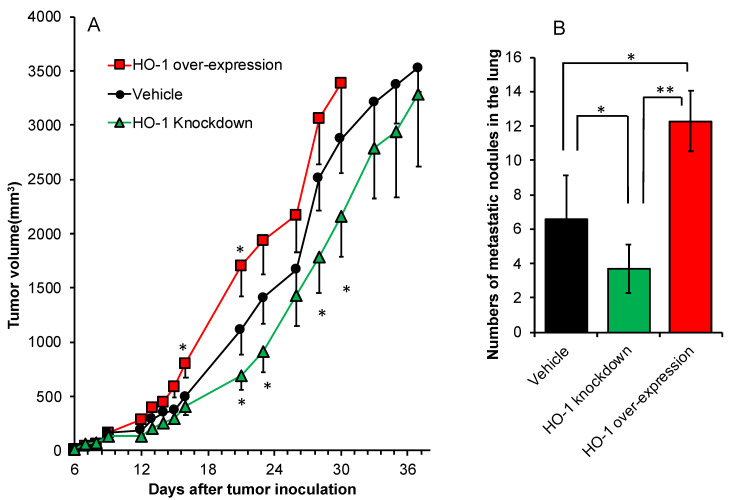
In vivo tumor growth of C26 cells with different expression levels of HO-1 in Balb/c mice. Wide C26 cells (transfected with lentivirus, vehicle), as well as C26 cells with HO-1 over-expression and HO-1 knock-down, were used in this study. After tumor inoculation, tumor volumes were measured every 2–3 days (**A**). At 40 days after tumor inoculation, mice were killed, and the lungs were collected. Tumor nodules in the lung were identified macroscopically, and numbers of metastatic nodules and each diameter were counted (**B**). Data are mean ± SEM, *n* = 6–8. * *p* < 0.05; ** *p* < 0.01. See text for details.

We further investigated the role of HO-1 in tumor growth and progression. The above-described C26 cells with different HO-1 expression levels were inoculated s.c. in Balb/c mice, and the growth of the implanted tumor is shown in Figure 5A. Compared to vehicle C26 cells, more rapid and aggressive tumor growth was observed when HO-1 over-expressed C26 cells were inoculated, whereas apparent delay of tumor growth was found for HO-1 knock-down C26 cells (Figure 5A). Moreover, remarkably increased lung metastasis was found in this solid tumor model using HO-1 over-expressed C26 cells, and HO-1 knock-down C26 cells exhibited a lower potential of metastasis than wild C26 cells and HO-1 over-expressed C26 cells (Figure 5B).

### 3.5. The Increase in HO-1 Level in the Host Suppresses the Growth and Metastasis of C26 Solid Tumor

**Figure 6:**
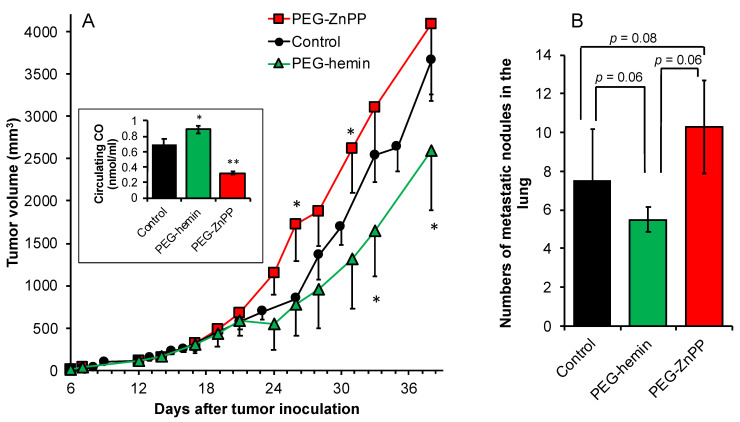
Effect of HO-1 inducer (PEG-hemin) and HO-1 inhibitor (PEG-ZnPP) on the growth of C26 solid tumor. Mouse colon cancer C26 solid tumor model using wild-type C26 cells was used in this study. One week before inoculation of C26 cells into mice, the mice were treated with PEG-hemin or PEG-ZnPP, both at 5 mg/kg (hemin or ZnPP equivalent) by intravenous injection (i.v.) thrice a week, which continued to 1 week after tumor inoculation. The tumor volumes were recorded every 2–3 days during the period of experiment (**A**). At 40 days after tumor inoculation, mice were killed, and the lungs were collected. Tumor nodules in the lung were identified macroscopically, and numbers of metastatic nodules and each diameter were counted (**B**). Data are mean ± SEM, *n* = 6–8. * *p* < 0.05; ** *p* < 0.01. See text for details.

To elucidate the effect of HO-1 expression in the host on the progression of the tumor, we induced or suppressed the HO-1 expression/activity by using an HO-1 inducer, PEG-hemin, which is a nanoformulation of the typical HO-1 inducer hemin [21], or HO-1 inhibitor PEG-ZnPP, which is a polymeric micellar formation of the competitive HO-1 inhibitor ZnPP [32]. Our previous studies showed that 5 mg/kg of PEG-hemin or PEG-ZnPP could significantly induce the expression or suppression of the activation of HO-1, respectively [22,32]. In line with the previous findings, we used the same dose of 5 mg/kg in this study, by which significantly increased or decreased HO-1 activity as described as circulating CO was achieved (inset of Figure 6A). When HO-1 was elevated in the host by PEG-hemin treatment, significant suppression of the tumor growth was found after the inoculation of C26 cells into the host (Figure 5A). On the contrary, increased tumor growth was observed when HO-1 activity in the host was inhibited (Figure 6A). In parallel with these findings, increased and decreased numbers of lung metastasis were found for the host with elevated and lowered HO-1 activities, respectively, though no statistical difference was observed (Figure 6B).

## 4. Discussion

CO is a major product of HO-1-catalyzed heme degradation [1], and it serves as a key molecule for the antioxidant and antiapoptotic functions of HO-1 [16]. It has been reported from different laboratories that exogenous administration of CO inhibits apoptosis in various cells, including fibroblasts, endothelial cells, and vascular smooth muscle cells [25,26,27]. Liu et al. also showed that survival in cultured vascular cells is mediated by the production of CO because the cytoprotection obtained from HO-1 is reversed by the CO scavenger, hemoglobin [27]. Moreover, a low concentration of CO can protect against hyperoxic lung injury in vivo [28]. More recently, we also reported that delivery of CO using a nano-designed CO donor to the liver remarkably improved the outcome of acetaminophen-evoked liver injury [35]. Thus, it is reasonable to suggest that CO produced from HO-1 in tumors confers the tumor cells protection against various attacks from the host, for example, ROS from infiltrated macrophages. With the growth and progression of the tumor, the tumor needs more and more of such a defense for its rapid growth. Consequently, HO-1 expression is induced highly and consistently, with a result of the gradually increased production of CO. This notion was supported by our results that CO production in the blood increased along with tumor growth and increased HO activity, as evidenced in two different tumor models (Figure 2 and Figure 3).

To further support our notion of the positive role of HO-1 in tumor growth, we established colon cancer C26 cell line with high HO-1 expression as well as HO-1 knock-down cell line (Figure 4A) and investigated their characteristic functions both in vitro and in vivo. As expected, HO-1 knock-down cells showed more vulnerability to anticancer drugs as well as ROS compared to parental C26 cells, whereas high HO-1 expression rendered the cells more resistant to these treatments (Figure 4B,C). When HO-1-over-expressing C26 cells were implanted into the mice, remarkably promoted tumor growth was observed, whereas apparently delayed tumor growth appeared in the HO-1 knock-down C26-cell-implanted model (Figure 5A). Moreover, lung metastasis exhibited similar trends, compared to parental C26 cells, HO-1-over-expressed C26 cells resulted in more lung metastasis, whereas HO-1 knock-down C26 cells showed significantly decreased numbers of metastatic nodules in the lung (Figure 5B). These findings are consistent with many previous reports, which suggested HO-1 is essential for tumor growth, progression, and metastasis [11,36,37,38,39]. However, there were also studies showing that HO-1 over-expression may induce tumor cell death and suppress tumor growth [40,41]. We consider this is at least partly due to the expression levels of HO-1. For example, in the present study, HO-1-over-expressed C26 cells showed a 1.5–2 times higher HO-1 expression than parental cells (Figure 4A). However, further HO-1 expression may result in the accumulation of heme metabolites bilirubin, CO, and free iron, which may induce cytotoxicity and induce cell death [42], in which free-iron-induced ferroptosis plays an important role [23,43]. The anticancer effects of CO-releasing molecules have also been reported in many studies [44]. Further studies are warranted to elucidate this issue.

Regarding the antioxidative, cell-protective role of HO-1, it has been widely understood that HO-1 is a critical protective molecule against ROS-related diseases and inflammation, as well as cancer [2,16,45]. Many cancer prevention agents fulfill their functions through the induction of HO-1 [30,46,47,48]. Namely, upregulating HO-1 levels in the hosts is an effective tool for suppressing carcinogenesis. Our present study confirmed this notion. When HO-1 levels in the mice were elevated by HO-1 inducer PEG-hemin, which was confirmed by the significantly increased circulation CO concentrations (inset of Figure 6A), tumor growth after implanting C26 cells showed apparent delay with decreased numbers of lung metastases compared to the mice without PEG-hemin pretreatment (Figure 6). In contrast, inhibition of HO-1 levels in the mice by PEG-ZnPP resulted in rapidly increased tumor growth as well as an increase in lung metastasis (Figure 6).

Taken together, the expression dynamics or balance of HO-1 in the host and cancer cells is a critical issue for the occurrence and progression of cancer. Basically, the induction of HO-1 in healthy cells and inhibition of HO-1 in cancer cells are desired events. However, it should be noted that the effects of HO-1 and its metabolites may vary depending on the doses/concentrations. Namely, the induction of HO-1 in the physiological ranges will exhibit protective effects. Excess levels/amounts of HO-1 or its metabolites may trigger opposite detrimental effects to the cells [23,43,44], which is another aspect of HO-1 that may become a therapeutic approach for cancer if it is properly manipulated.

## 5. Conclusions

The findings described in this study strongly suggest that the balance of HO-1 levels in the host and the tumor cells is essential for the occurrence, progression, and prognosis of cancer. The maintenance of acceptably high HO-1 levels in the host is a great benefit for protecting the host against various stresses, which is favorable for cancer prevention and suppression of tumor progression, resulting in better prognosis and outcomes for cancer patients. While tumor cells may also benefit from HO-1 expression against attacks from the host, consequently showing rapid growth, suppression of HO-1 in tumor cells may thus become a therapeutic strategy for cancer.

## Data Availability

The data presented in this study are available on request from the corresponding author.

## Supplementary Materials

The following are available online at https://www.mdpi.com/article/10.3390/jpm11121340/s1, Figure S1: CO production in rat AH136B ascitic tumor model. CO production in the blood of rats bearing AH136B ascitic tumor was measured by using NO to release CO bound to hemoglobin, which showed that 2 h incubation with NO reached a peak release of CO (A). A linear change of CO concentration was observed along with the concentration of ascites (B).

## References

[1] Schacter B.A. (1988). Heme catabolism by heme oxygenase: Physiology, regulation, and mechanism of action. Semin. Hematol..

[2] Maines M.D. (1988). Heme oxygenase: Function, multiplicity, regulatory mechanisms, and clinical applications. FASEB J..

[3] Baranano D.E., Rao M., Ferris C.D., Snyder S.H. (2002). Biliverdin reductase: A major physiologic cytoprotectant. Proc. Natl. Acad. Sci. USA.

[4] McCoubrey W.K., Huang T.J., Maines M.D. (1997). Isolation and characterization of a cDNA from the rat brain that encodes hemoprotein heme oxygenase-3. Eur. J. Biochem..

[5] Tenhunen R., Marver H.S., Schmid R. (1970). The enzymatic catabolism of hemoglobin: Stimulation of microsomal heme oxygenase by hemin. J. Lab. Clin. Med..

[6] Shibahara S. (1988). Regulation of heme oxygenase gene expression. Semin. Hematol..

[7] Keyse S.M., Tyrrell R.M. (1989). Heme oxygenase is the major 32-kDa stress protein induced in human skin fibroblasts by UVA radiation, hydrogen peroxide, and sodium arsenite. Proc. Natl. Acad. Sci. USA.

[8] Mitani K., Fujita H., Fukuda Y., Kappas A., Sassa S. (1993). The role of inorganic metals and metalloporphyrins in the induction of haem oxygenase and heat-shock protein 70 in human hepatoma cells. Biochem. J..

[9] Motterlini R., Foresti R., Bassi R., Calabrese V., Clark J.E., Green C.J. (2000). Endothelial heme oxygenase-1 induction by hypoxia. Modulation by inducible nitric oxide synthase and S-nitrosothiols. J. Biol. Chem..

[10] Foresti R., Clark J.E., Green C.J., Motterlini R. (1997). Thiol compounds interact with nitric oxide in regulating heme oxygenase-1 induction in endothelial cells. Involvement of superoxide and peroxynitrite anions. J. Biol. Chem..

[11] Doi K., Akaike T., Fujii S., Tanaka S., Ikebe N., Beppu T., Shibahara S., Ogawa M., Maeda H. (1999). Induction of haem oxygenase-1 by nitric oxide and ischaemia in experimental solid tumours and implications for tumour growth. Br. J. Cancer.

[12] Sato K., Balla J., Otterbein L., Smith R.N., Brouard S., Lin Y., Csizmadia E., Sevigny J., Robson S.C., Vercellotti G. (2001). Carbon monoxide generated by heme oxygenase-1 suppresses the rejection of mouse-to-rat cardiac transplants. J. Immunol..

[13] Wagner M., Cadetg P., Ruf R., Mazzucchelli L., Ferrari P., Redaelli C.A. (2003). Heme oxygenase-1 attenuates ishcemia/reperfusion-induced apoptosis and improves survival in rat renal allografts. Kidney Int..

[14] Agarwal A., Balla J., Alam J., Croatt A.J., Nath K.A. (1995). Induction of heme oxygenase in toxic renal injury: A protective role in cisplatin nephrotoxicity in the rat. Kidney Int..

[15] Lee T.S., Chau L.Y. (2002). Heme oxygenase-1 mediates the anti-inflammatory effect of interleukin-10 in mice. Nat. Med..

[16] Fang J., Akaike T., Maeda H. (2004). Antiapoptotic role of heme oxygenase (HO) and the potential of HO as a target in anticancer treatment. Apoptosis.

[17] Deininger M.H., Meyermann R., Trautmann K., Duffner F., Grote E.H., Wickboldt J., Schluesener H.J. (2000). Heme oxygenase (HO)-1 expressing macrophages/microglial cells accumulate during oligodendroglioma progression. Brain Res..

[18] Maines M.D., Abrahamsson P.A. (1996). Expression of heme oxygenase-1 (HSP32) in human prostate: Normal, hyperplastic, and tumor tissue distribution. Urology.

[19] Goodman A.I., Choudhury M., da Silva J.L., Schwartzman M.L., Abraham N.G. (1997). Overexpression of the heme oxygenase gene in renal cell carcinoma. Proc. Soc. Exp. Biol. Med..

[20] Tsuji M.H., Yanagawa T., Iwasa S., Tabuchi K., Onizawa K., Bannai S., Toyooka H., Yoshida H. (1999). Heme oxygenase-1 expression in oral squamous cell carcinoma as involved in lymph node metastasis. Cancer Lett..

[21] Sahoo S.K., Sawa T. (2002). Pegylated zinc protoporphyrin: A water-soluble heme oxygenase inhibitor with tumor-targeting capacity. Bioconjugate Chem..

[22] Fang J., Sawa T., Fang J., Tanaka S., Miyamoto Y., Akaike T., Maeda H. (2003). In vivo antitumor activity of pegylated zinc protoporphyrin: Targeted inhibition of heme oxygease in solid tumor. Cancer Res..

[23] Chiang S.K., Chen S.E., Chang L.C. (2019). A Dual Role of Heme Oxygenase-1 in Cancer Cells. Int. J. Mol. Sci..

[24] Luu Hoang K.N., Anstee J.E., Arnold J.N. (2021). The Diverse Roles of Heme Oxygenase-1 in Tumor Progression. Front. Immunol..

[25] Petrache I., Otterbein L.E., Alam J., Wiegand G.W., Choi A.M. (2000). Heme oxygenase-1 inhibits TNF-alpha-induced apoptosis in cultured fibroblasts. Am. J. Physiol. Lung Cell Mol. Physiol..

[26] Brouard S., Otterbein L.E., Anrather J., Tobiasch E., Bach F.H., Choi A.M., Soares M.P. (2000). Carbon monoxide generated by heme oxygenase 1 suppresses endothelial cell apoptosis. J. Exp. Med..

[27] Liu X.M., Chapman G.B., Peyton K.J., Schafer A.I., Durante W. (2002). Carbon monoxide inhibits apoptosis in vascular smooth muscle cells. Cardiovasc. Res..

[28] Otterbein L.E., Mantell L.L., Choi A.M. (1999). Carbon monoxide provides protection against hyperoxic lung injury. Am. J. Physiol..

[29] Yin H., Fang J., Liao L., Maeda H., Su Q. (2014). Upregulation of heme oxygenase-1 in colorectal cancer patients with increased circulation carbon monoxide levels, potentially affects chemotherapeutic sensitivity. BMC Cancer.

[30] Fang J., Seki T., Tsukamoto T., Qin H., Yin H., Liao L., Nakamura H., Maeda H. (2013). Protection from inflammatory bowel disease and colitis-associated carcinogenesis with 4-vinyl-2,6-dimethoxyphenol (canolol) involves suppression of oxidative stress and inflammatory cytokines. Carcinogenesis.

[31] Dong X., Li Z., Wang W., Zhang W., Liu S., Zhang X., Fang J., Maeda H., Matsukura M. (2011). Protective effect of canolol from oxidative stress-induced cell damage in ARPE-19 cells via an ERK mediated antioxidative pathway. Mol. Vis..

[32] Fang J., Qin H., Seki T., Nakamura H., Tsukigawa K., Shin T., Maeda H. (2011). Therapeutic potential of pegylated hemin for reactive oxygen species-related diseases via induction of heme oxygenase-1: Results from a rat hepatic ischemia/reperfusion injury model. J. Pharmacol. Exp. Ther..

[33] Mosmann T. (1983). Rapid colorimetric assay for cellular growth and survival: Application to proliferation and cytotoxicity assay. J. Immunol. Methods.

[34] Mohammad J., Singh R.R., Riggle C., Haugrud B., Abdalla M.Y., Reindl K.M. (2019). JNK inhibition blocks piperlongumine-induced cell death and transcriptional activation of heme oxygenase-1 in pancreatic cancer cells. Apoptosis.

[35] Song B., Zhang C., Hu W., Guo C., Xia Z., Hu W., Qin M., Jiang W., Lv J., Xu D. (2021). Nano-designed carbon monoxide donor SMA/CORM2 exhibits protective effect against acetaminophen induced liver injury through macrophage reprograming and promoting liver regeneration. J. Control. Release.

[36] Tanaka S., Akaike T., Fang J., Beppu T., Ogawa M., Tamura F., Miyamoto Y., Maeda H. (2003). Antiapoptotic effect of heme oxygenase-1 induced by nitric oxide in experimental solid tumor. Br. J. Cancer.

[37] Hall R., Malia R.G. (1991). Basic Haematological Practice. Medical Laboratory Haematology.

[38] Tsai J.R., Wang H.M., Liu P.L., Chen Y.H., Yang M.C., Chou S.H., Cheng Y.J., Yin W.H., Hwang J.J., Chong I.W. (2012). High expression of heme oxygenase-1 is associated with tumor invasiveness and poor clinical outcome in non-small cell lung cancer patients. Cell Oncol..

[39] Castruccio Castracani C., Longhitano L., Distefano A., Di Rosa M., Pittalà V., Lupo G., Caruso M., Corona D., Tibullo D., Li Volti G. (2020). Heme Oxygenase-1 and Carbon Monoxide Regulate Growth and Progression in Glioblastoma Cells. Mol. Neurobiol..

[40] Zou C., Zhang H., Li Q., Xiao H., Yu L., Ke S., Zhou L., Liu W., Wang W., Huang H. (2011). Heme oxygenase-1: A molecular brake on hepatocellular carcinoma cell migration. Carcinogenesis.

[41] Lin C.W., Shen S.C., Hou W.C., Yang L.Y., Chen Y.C. (2008). Heme oxygenase-1 inhibits breast cancer invasion via suppressing the expression of matrix metalloproteinase-9. Mol. Cancer Ther..

[42] Gamage S.M.K., Lee K.T.W., Dissabandara D.L.O., Lam A.K., Gopalan V. (2021). Dual role of heme iron in cancer; promotor of carcinogenesis and an inducer of tumour suppression. Exp. Mol. Pathol..

[43] Kwon M.Y., Park E., Lee S.J., Chung S.W. (2015). Heme oxygenase-1 accelerates erastin-induced ferroptotic cell death. Oncotarget.

[44] Tien Vo T.T., Vo Q.C., Tuan V.P., Wee Y., Cheng H.C., Lee I.T. (2021). The potentials of carbon monoxide-releasing molecules in cancer treatment: An outlook from ROS biology and medicine. Redox Biol..

[45] Fang J., Seki T., Maeda H. (2009). Therapeutic strategies by modulating oxygen stress in cancer and inflammation. Adv. Drug Deliv. Rev..

[46] Kabel A.M., Ashour A.M., Ali D.A., Arab H.H. (2021). The immunomodulatory effects of topiramate on azoxymethane-induced colon carcinogenesis in rats: The role of the inflammatory cascade, vascular endothelial growth factor, AKT/mTOR/MAP kinase signaling and the apoptotic markers. Int. Immunopharmacol..

[47] Gupta N., Verma K., Nalla S., Kulshreshtha A., Lall R., Prasad S. (2020). Free Radicals as a Double-Edged Sword: The Cancer Preventive and Therapeutic Roles of Curcumin. Molecules.

[48] Elsherbiny N.M., Eisa N.H., El-Sherbiny M., Said E. (2020). Chemo-preventive effect of crocin against experimentally-induced hepatocarcinogenesis via regulation of apoptotic and Nrf2 signaling pathways. Environ. Toxicol. Pharmacol..

