# Supplementary material for: Expression Dynamics of Heme Oxygenase-1 in Tumor Cells and the Host Contributes to the Progression of Tumors
Source: J Pers Med. 2021 Dec 9;11(12):1340. doi: 10.3390/jpm11121340 (PMC8704574; doi:10.3390/jpm11121340)
Supplement: Supplementary file 1 [file jpm-11-01340-s001.zip › jpm-1474693-supplementary.pdf]

Supplementary data Figure S1

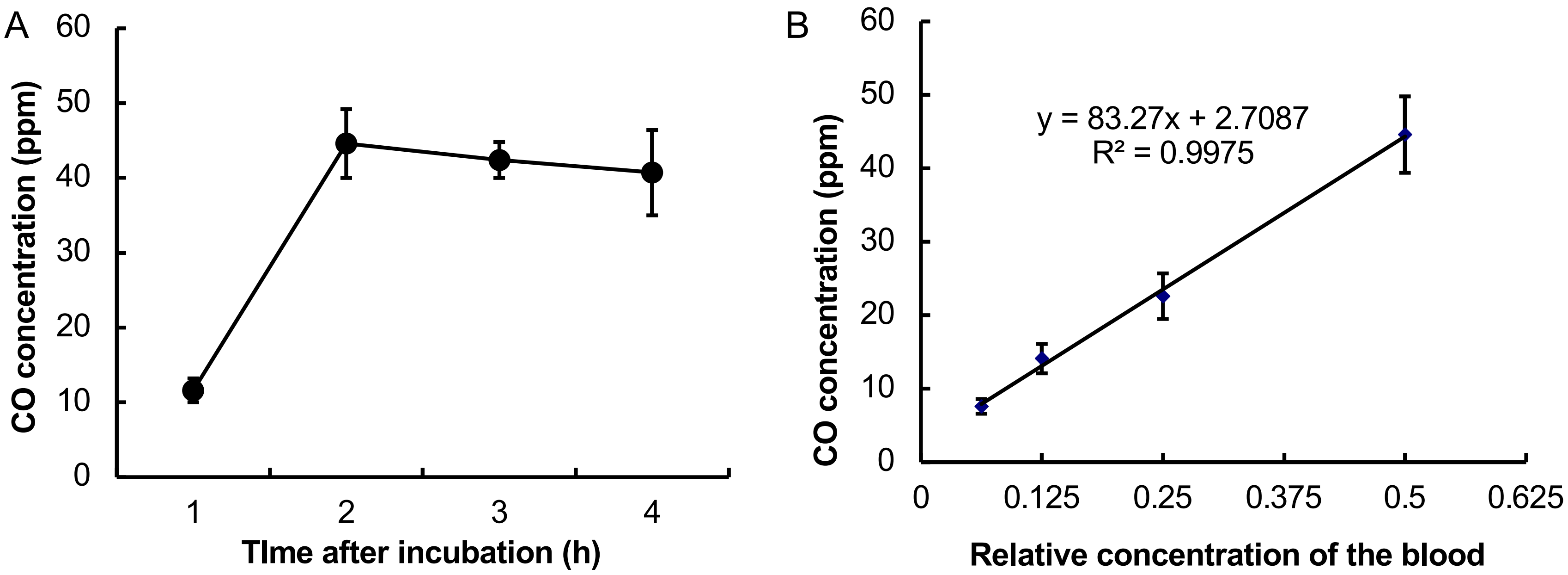

Figure S1. CO production in rat AH136B ascitic tumor model. CO production in the blood of rats bearing AH136B ascitic tumor was measured by using NO to release CO bound to hemoglobin, which showed that 2 h incubation with NO reached a peak release of CO (A). A linear change of CO concentration was observed along with the concentration of ascites (B).
